# Perceived Benefits and Barriers to Family Planning Education among Third Year Medical Students

**DOI:** 10.3885/meo.2008.Res00250

**Published:** 2008-04-24

**Authors:** Kimberly G. Smith, Melissa L. Gilliam, Mathieu Leboeuf, Amy Neustadt, Debra Stulberg

**Affiliations:** * Department of Obstetrics and Gynecology, University of Chicago, Chicago, IL 60637; † Department of Obstetrics and Gynaecology, Laval University, Québec City, Québec, Canada

**Keywords:** Medical student education, Professionalism, Family planning, Contraception, Abortion

## Abstract

**Purpose:**

The purpose of the current study is to explore third- year medical students’ interest in learning about family planning, exposure to family planning (contraception and abortion) and perceived barriers and benefits to family planning education in their obstetrics and gynecology rotation.

**Method:**

We conducted four focus groups with 27 third-year medical students near the end of their rotation in obstetrics and gynecology.

**Results:**

Students desired education in family planning but perceived limited exposure during their rotation. Most students were aware of abortion but lacked factual information and abortion procedural skills. They felt systemic and faculty-related barriers contributed to limited exposure. Students discussed issues such as lack of time for coverage of contraception and abortion in the curricula and rotation itself. Perceived benefits of clinical instruction in family planning included increased knowledge of contraceptive management and abortion the ability to care for and relate to patients, opportunity for values clarification, and positive changes in attitudes towards family planning.

**Conclusions:**

Medical students who desire full education in family planning during their obstetrics and gynecology rotation may face barriers to obtaining that education. Given that many medical students will eventually care for reproductive-age women, greater promotion of opportunities for exposure to family planning within obstetrics and gynecology rotations is warranted.

The Charter on Medical Professionalism identifies commitment to professional competence as one of the key professional responsibilities of physicians.^1^ According to the Association of Professors of Gynecology and Obstetrics (APGO), medical students “must be able to explain methods of pregnancy termination, identify potential complications, and provide non-directive counseling to patients surrounding pregnancy options.”^2^ Similarly, medical professionalism responsibilities endorsed by the Association of American Medical Colleges (AAMC) require that future physicians obtain the competencies needed to provide quality care to their patients.^1^ Most medical students will eventually care for reproductive-age women. According to a recent Gallup poll, 56% of women rely on health care providers for information about contraception. Similarly, family planning services (i.e., contraception and abortion) are among the most common services received by women of reproductive age.^3^ While most medical students select non-obstetrics and gynecology residencies,^4^ the majority choose fields in which they will routinely encounter women's reproductive health concerns, including family medicine, emergency medicine, and internal medicine. Thus, educational exposure to family planning, including contraceptive counseling and management and abortion, is necessary for medical students’ professional competence.

However, there appear to be significant gaps in medical student education on family planning and sexual health topics, leaving many physicians ill-equipped to manage their patients’ family planning needs.^5^ Indeed, previous research reveals medical students often have inaccurate knowledge about important reproductive health issues.^6^ Studies examining the acceptability and effectiveness of women's health electives and/or student-initiated courses with a family planning component have found that such courses resolve previous misunderstandings about abortion among medical students and enhance their learning of family planning.^7^ In addition, in a study by Epsey,^8^ 90% of students who participated in a family planning elective stated they would recommend the experience to other students. Of those who reported a change in attitude about abortion as a result of the elective, 94% became more supportive of access to abortion services. Others have examined personal attitudes and opinions among medical students toward family planning options and their intention to provide family planning services during their future medical practice.^7^,^9^–^11^ However, little is known about the level of exposure that occurs during obstetrics and gynecology rotations, barriers to such exposure, or the perceived value of training in family planning among students. We therefore undertook a qualitative study to explore students’ desire to learn about family planning, their level of exposure to contraception and abortion and perceived benefits and barriers to family planning education during their mandatory obstetrics and gynecology rotation. The paper offers suggestions for future research and concludes with a number of recommendations for improving medical student education in family planning within the obstetrics and gynecology rotation to be consistent with medical professional organizations’ requirements and guidelines.

## Method

We used focus groups in this study to capture rich data on medical students’ opinions about their exposure to family planning, perceived benefits and barriers to family planning education. Qualitative methodology is an important tool for gathering information on topics that have not been extensively researched and to develop themes and identify key issues for future research. The open-ended group format allows participants to share ideas with one another in order to bring up new issues, views, or insights not anticipated by the researchers and to direct the discussion toward issues about which students feel most strongly.^12^ Focus groups or group interviews also help participants express views about sensitive topics such as abortion that they might not express if interviewed individually. This study focuses on third-year medical students because the institution at which the study took place requires medical students to rotate in obstetrics and gynecology in their third year.

**Subjects** - We conducted four focus groups with 27 third-year medical students at a university in Chicago with a racially and ethnically diverse student body, a post-residency fellowship in family planning, and a family planning clinic with abortion and contraceptive services. All third year medical students rotating in obstetrics and gynecology at the university between January and June of 2005 were eligible to participate in the study. Students were informed of the study and invited to participate by the investigators during a scheduled weekly lecture toward the end of their obstetrics and gynecology rotation. Students were informed that participation in the study was voluntary and would have no effect on their end-of-rotation student evaluation. No participants were excluded based on sex, race, ethnicity, or religion. Students were not compensated for their involvement in the study. No other incentives were provided to the students for participation. Informed consent was obtained from each participant at the beginning of focus group sessions.

**Procedures** - Focus group sessions were conducted by the co-investigator (ML), a family planning fellow trained in qualitative research methods and interviewing, using a predetermined script with open-ended questions and set probes to help stimulate conversation. Students were asked about interest in family planning education, level of exposure to family planning (contraceptive methods, management and counseling, abortion procedures, public health and legal aspects of family planning) during the rotation, mode of training (lectures, readings, clinical encounters), if and how the experience influenced their beliefs, attitudes, and knowledge of family planning, and perceived opportunities for and barriers to exposure. Most focus groups included both females and males. All focus groups were conducted in English and lasted approximately 60 minutes. Participants also completed a demographic questionnaire. This study was approved by the university's institutional review board.

**Data analysis** - All focus group sessions were audio-taped, transcribed verbatim, and reviewed for accuracy. An initial codebook of broad themes based on the original research questions with operational definitions was developed to code the data. Following in-depth qualitative data analysis training, one independent coder coded the interviews in ATLAS/ ti, a qualitative software program for coding, text retrieval, and data management and analysis.^13^ A second independent coder reviewed the initial coding to ensure codes were being used accurately. Overall, there was high agreement in the use of codes; a quantitative measure for agreement, however, was not computed. The few instances of disagreement in coding were resolved through discussion among researchers.^14^ Codes and transcripts were reviewed to identify additional codes and salient themes not anticipated by the researchers. The final codes included contraception and abortion education, knowledge and attitude changes related to contraception and abortion, and coverage of contraception and abortion. Codes, specific phrases, sentences, and ideas were then aggregated into conceptual categories and important themes. Salient themes presented in this paper are those for which there was endorsement within and across focus groups. We use descriptive statistics to characterize the study sample and representative quotations to illustrate our findings.

**Table 1.** Descriptive Characteristics of the Study Sample (N = 27)

	N	%
Sex		
Women	13	48.2
Men	14	51.9
Place of birth		
Rural	3	12.0
Suburban	14	56.0
Urban	8	32.0
Race/Ethnicity		
African American	1	3.9
Asian	5	19.2
Caucasian	13	50.0
Caucasian, Hispanic	1	3.9
Hispanic	5	19.2
Other	1	3.9
Religion		
Atheist	1	4.0
Catholic	9	36.0
Christian (other)	7	28.0
Jewish	2	8.0
Hindu	2	8.0
Unitarian	1	4.0
None	1	4.0
N/A	2	8.0
Intended Specialty		
Emergency Medicine	3	3.7
Family Practice/Internal Medicine	3	3.7
Obstetrics and gynecology	2	7.4
Surgery	5	18.5
Other (e.g., Pediatrics, Pathology)	8	44.5
Undecided	6	22.2
Previous Work in Family Planning Clinic		
No	23	85.2
Yes	4	14.8

## Results

**Subject characteristics** - Except for one student, the entire third-year medical school class participated in the study. A total of 27 third-year medical students participated in four focus groups. We did not collect information on the one medical student who chose not to participate in the study. Nearly equal numbers of men and women participated in the study. The majority of students had grown up in suburban communities. While half of participants self-identified as Caucasian, focus groups included students of other racial/ethnic groups, primarily Asian and Hispanic. Most indicated they were Catholic and/or Christian. All students were 18 years of age or older; most in their mid-20s to early 30s. A minority of students had previous exposure to family planning (14.8%). Approximately 7% were planning to enter obstetrics and gynecology residencies post medical school.

Four predominant themes emerged from the data. First, nearly all students expressed a genuine interest in being exposed to family planning and obtaining factual information during their obstetrics and gynecology rotation. Second, students pointed to significant gaps in their family planning education. While most possessed basic knowledge of abortion and felt they gained some medical knowledge on contraception, only a few had the opportunity to observe or participate in contraceptive and abortion counseling or abortion procedures or to learn about sociopolitical aspects of family planning. Third, students described systemic and faculty-related barriers to exposure. For example, students felt there was a lack of time for coverage of contraception and abortion in the curricula or rotation and limited effort among faculty to make them aware of the family planning clinic located at the university hospital. Fourth, students described perceived benefits of clinical instruction in family planning, such as increased knowledge of contraceptive management and abortion and ability to care for and relate to patients. We next discuss each of the four themes and provide quotes to illustrate the themes.

**Interest in family planning education** - Overall, students expressed genuine interest in gaining knowledge and experience in family planning during the rotation without regard to their future career intentions and opinions about family planning topics. Many endorsed the idea that everyone knows about the myths and controversies about abortion, but as future doctors they felt it was important to learn the facts. This theme was highlighted in the following comment:

*So, I've also learned a lot about, you know, things like partial birth abortion, I was very uneducated about that. And, most people seem to think that it's something different from what it really is, so it was a very good experience to have. Even though I don't think I will be providing that sort of service, I'm glad I know more about it.*

There was widespread agreement that most students were aware of abortion but lacked factual information. A typical comment was as follows: “Even though you always hear about it [abortion], you don't know the specifics, so I learned about the specifics.” Similarly, another student emphasized the importance of observing an abortion procedure to better understand medical decision-making around abortion: “And I think that more of us should have the opportunity to see that, to see what happens in an abortion, because there's so much political hoopla surrounding the whole issue, but I think that most people are very poorly educated about it and they don't really know what's going on medically.”

**Level of exposure in family planning** - When asked if they thought there were enough lectures and formal teaching opportunities on contraception and abortion and how they learned about contraception, most students described learning about the medical aspects of contraception through lectures and clinical experiences during the rotation. One student summarized their overall impression by stating: “I think contraception options have been very well covered. And I think that's important. And I know, certainly, a lot more about contraception that's available for people and how it works than I did prior (to my rotation), so that has been good.”

However, when asked if there were any gaps in their family planning education, many described limited or no opportunities to directly observe and participate in contraceptive counseling and management, as illustrated by the following comment: “I never saw the counseling side which… I would say the more important part is like how you would bring about the subject, how it's being presented and stuff.” Even those few students who did have an opportunity to participate in counseling felt they did not have a clear understanding of contraceptive management guidelines. When asked if there was good teaching on postpartum contraceptive management, one participant describes her limited knowledge of contraceptive management for women who are breastfeeding:

*… and I still don't have a clear understanding of it but I know that when [the resident] was saying-umm-find out if this patient is breastfeeding cause that's going to-umm-you know determine what kind of choices are available to her-umm-but that's probably the extent of it.*

Students also felt there were limited opportunities to observe and/or participate in abortion counseling. In the words of one participant, “I didn't get any [abortion exposure] and if there was a procedure being done I never saw any abortion counseling of any patients.” Yet many expressed interest in exposure to abortions if the opportunity had been presented, as this student stated: “I think that, if they were to have even a day we could, you know, see the patient prior or afterwards and sit in on a couple of procedures, I would have been more than happy to take part.”

In addition, when asked if they thought they were exposed to the social and legal aspects of contraception and abortion, given the controversial nature and public health orientation of the topic, most felt the legal, policy, and financial aspects of contraception and abortion were not well covered. When asked about over-the-counter access to emergency contraception, a hotly-debated issue at the time of the rotation, most students were unaware of the issue. One stated, “It [over-the-counter emergency contraception] never really came up in this rotation.” Students were also unfamiliar with an Illinois state law mandating private insurance companies to provide coverage for contraceptive drugs and devices and outpatient contraceptive services.^15^ The only student who had heard of the law said, “The first time I've heard about that was in a billboard on the highway.” When asked specifically about the lack of federal funding for abortion, most students were surprised to hear from their peers in the focus group that federal funding does not support abortion procedures: “That's kind of surprising. I was pretty sure they did some sort of federal funding”.

Most wished the rotation provided more opportunity to explore and clarify their personal values, stereotypes, prejudices, and feelings regarding abortion. Consequently, students felt they missed an important opportunity and that their knowledge and/or attitudes towards abortion and contraception were unaffected by the rotation. One said disappointedly, “I feel as ignorant as I did before.” Another student described her frustration in this way:

*No [the rotation has not influenced my view on contraception or abortion]. I mean, like we've been saying, we haven't been exposed to the most controversial things that are most likely to have an impact on you. We just see the same things we do in other specialties so were unmoved.*

**Barriers to exposure to family planning** - Students described systemic and faculty-related barriers to family planning experience. According to students, *systemic* barriers stemmed from lack of time due to work load and limitations in the core curriculum. A typical comment was “There's just so many things to cover… so many different niches in OB/GYN and important parts in each of them that, scheduling-wise, you might miss something ’cause you keep changing services.” Others felt that there was lack of sufficient time for family planning in the obstetrics and gynecology rotation itself: “It's probably just because of the way the rotation is set up or there's not specific times for you to have that [family planning] exposure I guess.” They expressed frustration with the lack of time available for coverage of contraception and abortion, as they felt the rotation may be their only opportunity to gain important training in family planning services. This theme is illustrated by the following comment: “As far as gynecological issues are concerned, this [clerkship] is kind of it [the only chance for experience].

With respect to *faculty*-related barriers, students felt efforts by faculty to promote opportunities for training and exposure at the university's family planning clinic were minimal and that the onus was largely on students to seek out such training. Some attributed this lack of promotion to protective efforts among faculty to shield them from “sensitive issues” and/or to a fear among faculty about discussing abortion openly. For example, one student stated:

*I think maybe there is a fear among-umm-maybe the faculty and residents of offering it because they know that it is such a you know-umm-people have such strong feelings of one side or the other that they don't want to-umm-put people in a position to have to maybe say no. That they don't feel like they would want to go. I'm not sure. I'm just speculating that you know maybe that's part of why it hasn't really been bought up. I'm not sure.*

Similarly, some students felt emergency contraception was not well discussed due to controversy surrounding its use. For example, one participant stated, “I think (emergency contraception is) important but I haven't had any experience with it here because it's kind of sensitive.”

When asked if they knew about the family planning clinic at the university, many students were unaware that the family planning clinic existed or that abortions were performed at the institution. A typical comment was “I had no idea that that's what was done at the Family Planning Clinic.” Interestingly, the majority of students came across the clinic unexpectedly, initially believing it provided infertility treatment: “When I went to the family planning clinic I thought it had to do with like infertility, and when I got there I found out that it was D & Cs [dilatation and curettage] and I thought, oh well, this will be interesting.” Another student, also mistaking it for an infertility clinic, went because one of the attending physicians asked if a student was interested in going:

*There's a little tiny box in our schedule that says optional family planning clinic on Thursday afternoons, and the only reason I went was because Dr. _______ asked, she said she wanted a student to go that week and would any of us be willing and I thought you know, infertility, cool, I'll go, and I got very surprised when I went there.*

**Perceived value and benefits of exposure** - Though only a few students described the amount of training received as sufficient, students who gained experience in family planning during the rotation discussed multiple personal and professional benefits, including enhanced knowledge of family planning topics, increased ability to care for and relate to patients, and positive changes in attitudes towards abortion and/or contraception.

When asked if they learned anything new about contraception or abortion, a few students described increased knowledge and understanding of abortion procedures and contraception options. One student stated: “I wasn't aware that there was other things like IUD [intrauterine device]. I mean I was aware but I learned more about IUD placements and you know the specifics of it. Umm-so basically [I learned] the risks and benefits of contraception and things like that of different contraception.” Another said that previously she had not understood how emergency contraception worked and now had a better understanding of how it differed from a medical abortion: “I think there's a very common misperception, even in very well educated people, that it's actually an abortion pill similar to RU486 [Mifepristone] rather than something that actually prevents conception so and I found that even with my own family and they're all college educated.”

While few in number, some students felt they benefited from an increased understanding of the legal issues surrounding abortion, as well as governmental policies that hinder women's ability to obtain the procedure. One said, “I've also learned a lot about, you know, things like partial birth abortion, I was very uneducated about that. And most people seem to think that it's something different from what it really is.” Similarly, another female student said, “I didn't even know that there was a ban after 24 weeks on abortion. I thought that was what the partial abortion was, a late term abortion, and it's not, it's totally different.”

In addition, students felt exposure to family planning led to increased confidence in their ability to relate to, empathize with, and care for patients in need of family planning services. One student described how his experience made him more comfortable discussing family planning issues with patients:

*It [contraception] always kind of seemed kind of an intimidating thing to approach… but once I sat down and read about it and looked at it probably from a gynecologist perspective it seems a little more doable. It seems a lot simpler to me. I think it was becoming-umm-comfortable with [side effects].*

When asked if they learned more about the social aspects of family planning, some students described an improved understanding of circumstances surrounding unintended pregnancy, patient/provider decision-making regarding abortion, the context of second trimester abortion, and the personal challenges and difficulties patients face in making decisions regarding abortion. Students felt that the patient/provider decision-making process in particular showed how issues surrounding unintended pregnancy and abortion are not always clear-cut and that often there is a “gray area” involved in such decisions: “After having friends who have gone through it and also-umm-seeing patients who have gone through it, you know unwanted pregnancies-umm-like you just can't throw out a blank statement and say you know it's [abortion] wrong or right.”

Finally, when asked if the rotation affected their attitudes towards family planning or led to a change in opinion about contraception or abortion, students who were exposed described a number of positive changes in attitudes toward various aspects of family planning. After seeing a high number of teen and unintended pregnancies, most students had stronger feelings about the importance of contraceptive management, contraceptive access, and pregnancy prevention. For example, one participant who became more supportive of women's access to contraception stated, “Making contraception more available would be a good idea… stopping a pregnancy before it happens.”

Some became more supportive of abortion. Changes in attitude towards abortion resulted from learning about medical decision-making for abortions and/or observing actual abortion procedures. One student said, “I've lost a little bit of the distaste for it, because I've seen how much it can be medically necessary.” Another student felt she better understood the context and reasons behind decisions that patients had to make particularly around second trimester abortions:

*Especially, you know, the second trimester abortions, that's what I thought was really going to affect me, but surprisingly enough, those, that's what I was the most okay with because almost every single woman who was undergoing a second trimester abortion was due to serious medical complications. You know, horrible fetal anomalies that were incompatible with life.*

Others described changes in attitude after being exposed to issues surrounding access to family planning services and reproductive health care among low-income and/or uninsured patients. One student acknowledged a difficult situation faced by one of her patients:

*The reason she was having this baby was she couldn't afford, she didn't have any prenatal care but she couldn't afford to have an abortion. She has-umm-one kid with a genetic illness, another kid with two developmental illnesses and… she's 20 years old. I'm not sure personally you know exactly how I stand with how it [abortion] should be funded, but I think it's definitely a problem.*

Even those who did not necessarily feel their attitude toward abortion changed as a result of the rotation felt they benefited from the opportunity to think about the issue more extensively and to reaffirm or clarify their own values regarding abortion. One said, “I definitely have to agree that abortion is much more immediate in my mind now. Not to say that the experience has changed my position, but something I think more clearly about”.

## Conclusions

Overall, students expressed genuine interest in gaining experience in family planning during the obstetrics and gynecology rotation. Most expected and desired specific opportunities for increased understanding of policy and medical issues involved in family planning, as they felt education and training among health care providers in family planning services were necessary to ensure women's reproductive rights and for the continuation of safe abortion provision. However, few were able to participate in and/or observe contraceptive and abortion counseling or abortion procedures or to learn about sociopolitical aspects of family planning. While poor coverage of family planning, particularly abortion, has been noted by others,^16^ our findings suggest systemic and faculty-related barriers may prevent medical students from observing abortion procedures and gaining knowledge of the legal, policy, and financial aspects of family planning.

Conversely, students who participated in the family planning service found it to be one of the most rewarding aspects of the obstetrics and gynecology rotation, as it allowed them to learn about contraceptive management and pregnancy options counseling and to gain insight into circumstances surrounding unintended pregnancy and pregnancy prevention. Learning more about the social and medical context of a patient's abortion decision-making provided opportunities for students to examine and clarify their personal values and beliefs about abortion. Consistent with previous research, additional positive outcomes included enhanced knowledge of medical, legal, and ethical issues surrounding abortion, positive changes in attitudes toward abortion and/or contraception, and increased comfort and confidence in caring for and empathizing with patients seeking pregnancy options counseling and/or abortion services in order to provide patient-centered care.^7^–^8^

This study has limitations. First, it is a qualitative study of students from a single medical school. It does not address the prevalence of these views and experiences at other medical institutions. However, this study was exploratory in nature and was conducted to gather rich data on insights, issues, and views among medical students on their family planning education in order to inform future research studies. Additionally, because the sample was diverse, it is likely that similar results would be found in other medical schools. Second, the moderator was a family planning fellow within the obstetrics and gynecology department, though not directly involved in the evaluation of students, which may have limited students’ ability to respond impartially to the questions or comfort in sharing their opinions. Students may have also felt pressure from their peers to respond to questions in a certain way. However, to minimize the chances of socially desirable responses, discomfort, or peer conformity, the moderator used a variety of approaches, such as employing a conversational approach to questioning, encouraging diverse perspectives and specific examples, emphasizing that all results would be kept confidential and de-identified, would not affect their school standing; he did not share his own personal opinions about family planning with the students. Most participants expressed their opinions and views in the focus groups. In only one of the larger focus groups, two participants did not express any of their views.

Ultimately, our study demonstrates that students at this institution perceived significant gaps in their medical education in the area of family planning. The obstetrics and gynecology rotation is a unique opportunity to teach important knowledge and skills in contraceptive management, pregnancy prevention, and the public health consequences of unintended pregnancy. Limited coverage of controversial policy-related issues misses critical early opportunities to assist students in exploring their own attitudes, opinions, and values about family planning and reproductive care. We recommend obstetrics and gynecology faculty work to raise awareness among medical students of existing family planning services in order to facilitate opportunities for training and personal growth. Faculty may consider informing students of the services at forums such as student orientations, lectures, and journal clubs, as our study highlights the perceived personal and professional benefits afforded to students through exposure to family planning which will serve them well as future physicians. This study can also inform future research. Findings can be used to develop a quantitative survey to assess whether the experiences of these medical students are mirrored at other medical schools. Future research could compare medical students’ experiences in family planning across the United States (US) by year or location (e.g., regions, urban/rural). Understanding the prevalence of these views and experiences among a national or regional sample of medical students could lead to significant improvements in family planning medical education and ensure future physicians in the US are adequately trained in reproductive care and aware of the public health aspects of family planning.
